# Multilayered Thin Films from Boronic Acid-Functional Poly(amido amine)s

**DOI:** 10.1007/s11095-015-1688-0

**Published:** 2015-04-08

**Authors:** Sry D. Hujaya, Johan F. J. Engbersen, Jos M. J. Paulusse

**Affiliations:** Department of Controlled Drug Delivery, MIRA Institute for Biomedical Technology and Technical Medicine, Faculty of Science and Technology, University of Twente, P.O. Box 217, 7500 AE Enschede, The Netherlands

**Keywords:** biodegradable polymers, chondroitin sulfate, layer-by-layer assembly, multilayered thin films, poly(vinyl alcohol)

## Abstract

**Purpose:**

To investigate the properties of phenylboronic acid-functional poly(amido amine) polymers (BA-PAA) in forming multilayered thin films with poly(vinyl alcohol) (PVA) and chondroitin sulfate (ChS), and to evaluate their compatibility with COS-7 cells.

**Methods:**

Copolymers of phenylboronic acid-functional poly(amido amine)s, differing in the content of primary amine (DAB-BA-PAA) or alcohol (ABOL-BA-PAA) side groups, were synthesized and applied in the formation of multilayers with PVA and ChS. Biocompatibility of the resulting films was evaluated through cell culture experiments with COS-7 cells grown on the films.

**Results:**

PVA-based multilayers were thin, reaching ~100 nm at 10 bilayers, whereas ChS-based multilayers were thick, reaching ~600 nm at the same number of bilayers. All of the multilayers are stable under physiological conditions *in vitro* and are responsive to reducing agents, owing to the presence of disulfide bonds in the polymers. PVA-based films were demonstrated to be responsive to glucose at physiological pH at the investigated glucose concentrations (10–100 mM). The multilayered films displayed biocompatibility in cell culture experiments, promoting attachment and proliferation of COS-7 cells.

**Conclusions:**

Responsive thin films based on boronic acid functional poly(amido amine)s are promising biocompatible materials for biomedical applications, such as drug releasing surfaces on stents or implants.

**Graphical Abstract:**
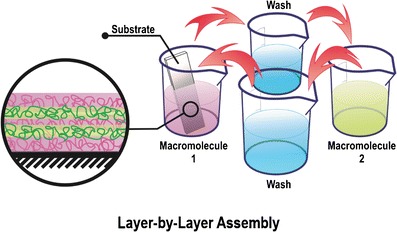
Layer-by-Layer Assembly

## Introduction

**Scheme 1:**
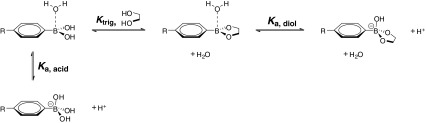
The equilibria of boronate ester formation and relevant Lewis acidity.

In the development of multi-responsive drug delivery systems, the reversible ester formation of boronic acid (BA) with diols (Scheme 1) has been of widespread interest (1,2). This interaction is considered quite special not only due to its specific coordinative covalent nature, but also due to the dynamic properties of the interaction (3,4). This chemistry is very relevant to the biomedical field as it takes place under physiological conditions, is highly sensitive to pH, and concerns diol-moieties which can be found in many biologically relevant compounds and macromolecules, most notably carbohydrates and sugars. Therefore, this ester formation has been widely exploited for development of blood glucose sensors (5–9), separation agents (10–12), and therapeutic delivery systems (hydrogels (13,14), micro- and nanoparticles (2,15,16)) for insulin (17–20), and other drugs (21–23). Phenylboronic acid, a more reactive derivative of alkylboronic acid in ester formation with diols, has been found to selectively inhibit tumor cells without much effect on non-tumorigenic cell lines (24).

The boronate ester formation has also been exploited to develop multilayered thin films (25–28). In contrast to most multilayer films formed by LbL technology, these films rely not on simple electrostatic interactions, but on the multi-responsive boronate ester formation, which—combined with the versatility of the layer-by-layer (LbL) dip-coating technique—make them a very promising approach to provide complex 3D structures (*e.g.*, implants, stents, prostheses) with multi-responsive drug releasing properties.

**Scheme 2:**
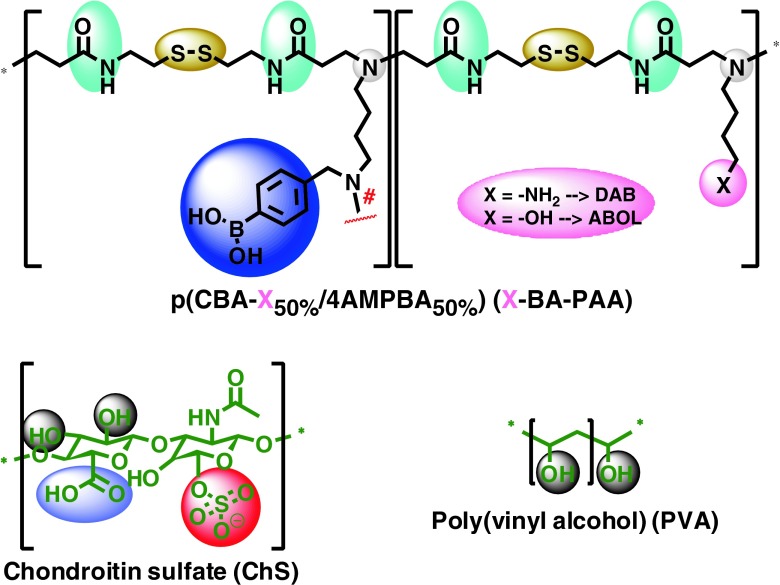
Chemical structures of DAB-BA-PAA, ABOL-BA-PAA, ChS, and PVA utilized as main components for multilayer formation. Control polymer DAB-Bn-PAA is similar to DAB-BA-PAA but with benzyl moiety instead of the phenylboronic acid moiety.

Two phenylboronic acid-functionalized poly(amido amine)s (BA-PAA) differing in the presence of primary amine (DAB-BA-PAA) or primary alcohol (ABOL-BA-PAA) side groups (Scheme 2) have been synthesized and studied for their properties in forming multilayered thin films with two different diol-containing macromolecules. The presence of disulfide bonds in the polymer main chain renders them responsive to reducing agents. The branched structure of the BA-PAA adds multivalency to strengthen interactions with the diol-containing counterpart and increases overall multilayer stability. However, unlike the poly(amido amine) dendrimers (PAMAM) with high degree of branching, the BA-PAAs may still maintain structural flexibility in solution—a feature that has been reported to increase their potency as gene carriers (29,30). The linear form of *ortho*-substituted ABOL-BA-PAA itself has been previously reported to self-assemble into nanoparticles capable of intracellular delivery of alizarin red S (a diol-containing small molecule) as model drug with the BA-PAA polymer (16), implying the possibility of achieving nanoparticle-releasing multilayered system.

The other multilayer components, poly(vinyl alcohol) (PVA) and chondroitin sulfate (ChS) are chosen to represent widely-studied diol-containing macromolecules that are in principle suitable for biomedical applications. PVA is a component of several US Food and Drug Administration (FDA)-approved products/biomedical devices (31,32), used in the preparation of adhesives, paper and packaging, textiles, toys, cosmetics, eye drops, embolization agents, and is commercially available in various molecular weights. ChS is a carbohydrate found in cartilage and bone extracellular matrix. It is a linear biopolymer of repeating *N*-acetyl-D-galactosamine and D-glucuronic acid disaccharide, and is often consumed as supplement for treatment of osteoarthritis (33–35).

In this report, the two BA-PAAs are paired with both PVA and ChS to form multilayers. The build-up profiles and properties of the resulting ensembles are described in relation to the underlying intermolecular interactions. As a control, DAB-Bn-PAA (*i.e.*, DAB-BA-PAA without the BA moiety, but rather a benzyl-moiety) was also synthesized and used to verify the role of BA-diol interactions. Scheme 2 depicts the chemical structures of the four main multilayer components. It is hypothesized that thin films from PAA polymers in combination with PVA and ChS will endow surfaces with biocompatibility. Moreover, incorporation of boronic acid moieties into the poly(amido amine) polymers is hypothesized to render the thin films glucose responsive.

## Materials and Methods

*N,N′*-Cystamine bisacrylamide (CBA, 99.9%) was purchased from Polysciences (Eppelheim, Germany). Sodium sulfate (Na_2_SO_4_, 99%) was purchased from Acros (Landsmeer, The Netherlands). 4-Amino-1-butanol (ABOL, 98.0%), *N*-Boc-1,4-diaminobutane (NBDAB, ≥97.0%), 4-formylphenylboronic acid (4FPBA, ≥95.0%), sodium borohydride (NaBH_4_, ≥98.0%), *N*-benzyl-1,4-butanediamine (BnDAB), calcium chloride (CaCl_2_, ≥93.0%), triethylamine (TEA, ≥99.0%), *tert*-butylamine (*t*BA, ≥99.5%), trifluoroacetic acid (TFA, ≥99.0%), sodium chloride (NaCl, ≥99.5%), Mowiol® 4–98, 6–98, 56–98 (PVA, ~27, ~47, ~195 kDa), Chondroitin 4-sulfate sodium salt from bovine trachea (ChS, ≤10% water), glucose (≥99.5%), and glutathione (≥98.0%) were purchased from Sigma-Aldrich (Zwijndrecht, The Netherlands). Sodium dihydrogen phosphate monohydrate (NaH_2_PO_4_.H_2_O, 99.0–102.0%), disodium hydrogen phosphate dihydrate (Na_2_HPO_4_.2H_2_O, 99.5%), citric acid (≥99%), and trisodium citrate dihydrate (≥99%) were purchased from Merck (Darmstadt, Germany). Solvents were of reagent grade and used without further purification unless otherwise noted. Milli-Q water (MΩ∙cm at 25°C) was obtained from a Synergy® water purification system (Millipore).

PBS buffer was prepared by dissolving 1.54 g of Na_2_HPO_4_.2H_2_O, 0.30 g of NaH_2_PO_4_.H_2_O, and 8.20 g of NaCl into 1.00 L of Milli-Q water and adjusting the pH to 7.4.

Citrate buffered saline (CBS buffer) pH 4, 5, and 6 were prepared by dissolving citric acid, trisodium citrate dehydrate and NaCl in the appropriate amounts in Milli-Q and adjusting the pH with HCl or NaOH.

^1^H NMR spectra were recorded on an AVANCE III-400 MHz NMR (Bruker, Wormer, The Netherlands) spectrometer.

UV characterization of multilayered thin films was performed in the dry state using a UV-2401 PC (Shimadzu, ‘s-Hertogenbosch, The Netherlands). Each film fabricated on UV-transparent 7.5 × 37 × 1 mm quartz glass (Ted Pella, Redding, USA) was measured in three different arbitrary positions. Absorbance scan was carried out in the 200–400 nm wavelength range. All data points were then corrected for baseline offset by subtracting the absorbance value at 400 nm from each data point. Relative absorbance values were obtained by normalizing each data point with the respective value at time 0.

AFM characterization was performed on a Multimode AFM (Bruker, Wormer, The Netherlands) with Nanoscope IV controller in contact mode using an MSCT cantilever with moderate spring constant of 0.5 N/m. Multilayered thin film samples were fabricated on single side polished silicon wafer (n-type, 525 μm thick, MESA+NanoLab, Enschede, The Netherlands) diced into 7.5 × 32 mm pieces.

Contact angle measurements were performed on a Krüss G10 (KRÜSS, Hamburg, Germany) contact angle measuring instrument.

Poly-D-lysine-coated 96 well plates (PDL-TCPS) for multilayer build-up for cell culture and transfection experiments were purchased from Greiner (Alphen aan den Rijn, The Netherlands).

COS-7 cells (European Collection of Animal Cell Cultures (ECACC) Catalogue No. 87021302) were grown in DMEM containing 4.5 g/L glucose and GlutaMAX™ (Invitrogen, Breda, The Netherlands) supplemented with 2% (*v/v*) PennStrepp (Lonza, Breda, The Netherlands) and 10% (*v/v*) fetal bovine serum (Lonza, Breda, The Netherlands).

Cell imaging was performed at 4×, 10×, 20×, and/or 40× objectives using EVOS digital inverted microscope (EMS, Wageningen, The Netherlands) equipped with RFP and DAPI light cubes for EthD-1 and Hoechst 33258 fluorescence imaging, respectively. EthD-1 and Hoechst 33258 was purchased from Invitrogen (Breda, The Netherlands) and Sigma-Aldrich, respectively.

Fluorescence intensity measurements were carried out in an Infinite M200 PRO plate reader (Tecan, Giessen, The Netherlands). AlamarBlue for cell viability measurements was purchased from Invitrogen (Breda, The Netherlands).

### Synthesis of 4-((4-aminobutylamino)methyl)phenylboronic Acid (4AMPBA)

For the synthesis of 4-((4-aminobutylamino)methyl)phenylboronic acid, 4-formylphenylboronic acid (3.12 g, 20.8 mmol) was mixed with *N*-Boc-1,4-diaminobutane (4.03 g, 20.8 mmol) in a round-bottom flask. The reactants were dissolved in 25 mL of methanol and stirred under nitrogen atmosphere at room temperature for at least 4 h to let the imine formation proceed to completion. To the brownish solution, sodium borohydride (0.79 g, 21.3 mmol) was then added portion-wise. The imine reduction was observed from the change in color of the solution to yellow, indicating shortening of double bond conjugation. This reaction was let to proceed for 2 h after which the remaining methanol was evaporated under reduced pressure, and the rest of the solid product mixture was dissolved in Milli-Q water and extracted with equal volume of chloroform for 6 times followed by one time backwashing. The chloroform phases were collected, and dried with Na_2_SO_4_. The water-free chloroform solution was then evaporated under reduced pressure, leaving the BOC-protected solid product behind. Molar yield: ~60%. The BOC-protected product was deprotected by first dissolving the product in 15 mL of methanol in a round-bottom flask. HCl gas (prepared by drop-wise addition of H_2_SO_4_ to NaCl) was then purged into the solution under nitrogen stream. The reaction was left to continue for at least an hour to proceed to completion. The volatile components in the solution were then evaporated to yield the product as a white powder in its HCl salt form. The product was further dried *in vacuo* and stored in the freezer. Yield: 87%. ^1^H-NMR (HCl salt, CD_3_OD, 400 MHz) δ (ppm) = 1.66 (q, 2H, NH_2_CH_2_C***H***_***2***_); 1.82 (q, 2H, NH_2_CH_2_CH_2_C***H***_***2***_); 2.97 (t, 2H, NH_2_C***H***_***2***_); 3.09 (t, 2H, ArCH_2_NHC***H***_***2***_); 4.21 (s, 2H, ArC***H***_***2***_NH); 7.48 (d, 2H, Ar***H***); 7.76 (d, 2H, Ar***H***).

### Synthesis of p(CBA-DAB_50%_/4AMPBA_50%_) (DAB-BA-PAA)

*N,N′*-Cystamine bisacrylamide (2.02 g; 7.76 mmol), *N*-Boc-1,4-diaminobutane (0.74 g; 3.88 mmol), and 4AMPBA (1.14 g; 3.88 mmol) were mixed in a brown polymerization flask using 2 mL of methanol/water 3/1 as solvent and containing 200 mM CaCl_2_ as catalyst based on recent report by Zintchenko *et al.* (36). Triethylamine (1.3 mL; 9.7 mmol) was also added to free the amines in 4AMPBA for Michael addition reaction. Polymerization was carried out under N_2_ atmosphere for 7 days at 70°C during which a gradual viscosity increase was observed. The polymerization was terminated by adding *tert*-butylamine (2.5 mL; 23.47 mmol) into the mixture and stirring at 70°C for 2 more days. After bringing the flask to room temperature, the solution was diluted and acidified to pH ~4 by addition of 4 M HCl and purified by ultrafiltration using a 1000 Da MWCO membrane. The purified polymer solution was then freeze-dried leaving white yellowish solid as the BOC-protected product in its HCl-salt form (2.1 g; 54% recovery). ^1^H-NMR characterization was performed to confirm complete conversion of terminal acrylate groups by reaction with *tert*-butylamine.

Deprotection was carried out with HCl gas as mentioned in the synthesis of the 4AMPBA monomer. After evaporation of volatile components, the solid was redissolved in water, brought to slightly acidic pH, filtered and further purified by ultrafiltration using a 1000 Da MWCO membrane. The purified polymer solution was finally freeze-dried to yield a light yellow solid as the final product in its HCl-salt form (0.92 g; 44% recovery). ^1^H-NMR characterization was performed to confirm complete deprotection. ^1^H NMR (D_2_O) δ (ppm) = 1.35 (s, 9H, (C***H***_***3***_)_***3***_R); 1.75 (m, 4H, C***H***_***2***_CH_2_NH_2_ & CH_2_C***H***_***2***_CH_2_N(BA)); 1.86 (m, 4H, C***H***_***2***_CH_2_CH_2_NH_2_ & C***H***_***2***_CH_2_CH_2_N(BA)); 2.79–2.83 (broad, 16H, C***H***_***2***_CONHRNHCOC***H***_***2***_ & C***H***_***2***_SSC***H***_***2***_); 3.05 (m, 4H, C***H***_***2***_NH_2_ and C***H***_***2***_NH(BA)); 3.28 (t, 4H, N(CH_2_)_3_C***H***_***2***_); 3.47–3.49 (broad, 16H, NCOCH_2_C***H***_***2***_NRC***H***_***2***_ and C***H***_***2***_CH_2_SSCH_2_C***H***_***2***_); 4.25 (2H, s, ***branched*** ArH-C***H***_***2***_-N-(CH_2_)_2_); 4.42 (2H, s, ArH-C***H***_***2***_-NH); 7.52 (2H, d, Ar***H***), 7.83 (2H, d, Ar***H***). Degree of BA functionalization was estimated at 53%, 19% of which reacted further through the benzylic amine to form branches.

### Synthesis of p(CBA-ABOL_50%_/4AMPBA_50%_) (ABOL-BA-PAA)

*N,N′*-Cystamine bisacrylamide (1.08 g; 4.17 mmol), 4-amino-1-butanol (0.19 g; 2.13 mmol), and 4AMPBA (0.62 g; 2.09 mmol) were mixed in a brown polymerization flask using 2.3 mL of methanol/water 3/1 as solvent and containing 200 mM CaCl_2_ as catalyst based on recent report by Zintchenko *et al.* (36). Triethylamine (1.5 mL; 10.7 mmol) was also added to deprotonate the amines in 4AMPBA for Michael addition reaction. Polymerization was carried out under N_2_ atmosphere for 2 days at 70°C during which a gradual viscosity increase was observed. The polymerization was terminated by adding excess of *tert*-butylamine (2.2 mL; 20.9 mmol) into the mixture and stirring at 70°C for 6 more days until no trace of acrylates was detected with ^1^H-NMR. After bringing the reaction mixture to room temperature, the solution was diluted with water and acidified to pH ~4 by addition of 4 M HCl and purified by ultrafiltration using a 1000 Da MWCO membrane. The purified polymer solution was then freeze-dried leaving white solid as the HCl-salt form (0.58 g; 31% recovery). ^1^H-NMR characterization was performed to confirm complete conversion of terminal acrylate groups by reaction with *tert*-butylamine and estimation of degree of polymerization. ^1^H NMR (D_2_O) δ (ppm) = 1.36 (s, 9H, (C***H***_***3***_)_***3***_R); 1.60 (m, 4H, CH_2_C***H***_***2***_CH_2_N(BA) & C***H***_***2***_CH_2_CH_2_OH); 1.78 (m, 4H, C***H***_***2***_CH_2_CH_2_N(BA) & C***H***_***2***_CH_2_OH); 2.70 (t, 8H, C***H***_***2***_CONHRNHCOC***H***_***2***_); 2.83 (t, 8H, C***H***_***2***_SSC***H***_***2***_); 3.09 (t, 2H, CH_2_CH_2_C***H***_***2***_N(BA)); 3.20 (t, 2H, C***H***_***2***_CH_2_CH_2_CH_2_OH); 3.29 (t, 2H, C***H***_***2***_CH_2_CH_2_CH_2_N(BA)); 3.35 – 3.58 (broad, 16H, C***H***_***2***_CH_2_SSCH_2_C***H***_***2***_ & C***H***_***2***_CH_2_CONHRNHCOCH_2_C***H***_***2***_); 3.61 (t, 2H, HOC***H***_***2***_); 4.25 (2H, s, ***branched*** ArH-C***H***_***2***_-N-(CH_2_)_2_); 4.31 (broad, 2H, s, ArH-C***H***_***2***_-NH); 7.50 (2H, d, Ar***H***), 7.83 (2H, d, Ar***H***). Degree of BA functionalization was estimated at 52%, 59% of which reacted further through the benzylic amine to form branches.

### Synthesis of p(CBA-DAB_50%_/BnDAB_50%_) (DAB-Bn-PAA)

The polymer DAB-Bn-PAA that serves as a negative control for the boronic acid activity of the DAB-BA-PAA and ABOL-BA-PAA polymers was synthesized by mixing *N,N′*-cystamine bisacrylamide (1.32 g; 5 mmol), *N*-Boc-1,4-diaminobutane (0.47 g; 2.5 mmol), and BnDAB (0.45 g; 2.5 mmol) in a brown polymerization flask using 2.6 mL of methanol/water 3/1 as solvent and containing 200 mM CaCl_2_ as catalyst based on recent report by Zintchenko *et al.* (36). Polymerization was carried out under N_2_ atmosphere for 2 days at 70°C during which a gradual viscosity increase was observed. The polymerization was terminated by adding *tert*-butylamine (1.8 mL; 16.9 mmol) into the mixture and stirring at 70°C for 2 more days until no trace of acrylates was detected with ^1^H-NMR. After bringing the flask into room temperature, the solution was diluted and acidified to pH ~4 by addition of 4 M HCl and purified by ultrafiltration using a 1000 Da MWCO membrane. The purified polymer solution was then freeze-dried leaving yellowish solid as the BOC-protected product in its HCl-salt form (1.5 g; 65% recovery). ^1^H-NMR characterization was performed to confirm complete conversion of terminal acrylate groups by reaction with *tert*-butylamine

Deprotection was carried out with HCl gas as described in the synthesis of the 4AMPBA monomer. After evaporation of volatile components, the solid was redissolved in slightly acidic water, though part of the solid was insoluble. The soluble portion was filtered and further purified by ultrafiltration using a 1000 Da MWCO membrane. The purified polymer solution was finally freeze-dried to yield the final product in its HCl-salt form as a brown solid (0.15 g; <10% recovery). ^1^H-NMR characterization was performed to confirm complete deprotection. ^1^H NMR (D_2_O) δ (ppm) = 1.36 (s, 9H, (C***H***_***3***_)_***3***_R); 1.76 (m, 4H, C***H***_***2***_CH_2_NH_2_ & CH_2_C***H***_***2***_CH_2_N(Bn)); 1.86 (m, 4H, C***H***_***2***_CH_2_CH_2_NH_2_ & C***H***_***2***_CH_2_CH_2_N(Bn)); 2.80 (t, 8H, C***H***_***2***_CONHRNHCOC***H***_***2***_); 2.84 (t, 8H, C***H***_***2***_SSC***H***_***2***_); 3.05 (m, 4H, C***H***_***2***_NH_2_ and C***H***_***2***_NH(Bn)); 3.28 (broad, 4H, N(CH_2_)_3_C***H***_***2***_); 3.38–3.8 (broad, 16H, NCOCH_2_C***H***_***2***_NRC***H***_***2***_ and C***H***_***2***_CH_2_SSCH_2_C***H***_***2***_); 4.25 (2H, s, ***branched*** ArH-C***H***_***2***_-N-(CH_2_)_2_); 4.41 (2H, s, ArH-C***H***_***2***_-NH); 7.52 (5H, s, Ar***H***). Degree of BA functionalization was estimated at 60%, 21% of which reacted further through the benzylic amine to form branches.

### Multilayered Thin Film Construction and Build-Up Profiles

Fresh BA-PAA solutions were prepared short before the start of multilayer build-up from the solid materials which had been re-lyophilized overnight to avoid weighing errors due to their hygroscopic properties. All BA-PAA solutions (2.0 mg/mL) were prepared in PBS buffer at pH 7.4 to avoid possible variations in pH.

Prior to the assembly, quartz or silicon wafer substrates (7.5 × 32 mm) were etched for 30 min in piranha acid to activate the surface, rinsed with copious amounts of Milli-Q water, and dried under N_2_ stream. These substrates were then dipped into BA-PAA solution (2.0 mg/mL in PBS buffer pH 7.4) for 5 min, transferred into washing solution containing PBS buffer for 1 min, dipped briefly in a large amount of Milli-Q water, transferred into ChS or PVA (2 mg/mL in Milli-Q water and PBS buffer pH 7.4, respectively) solution for 5 min, dipped into the second washing solution containing Milli-Q water or PBS buffer for 1 min, and finally followed by another brief dipping in Milli-Q. This cycle was repeated to reach the desired number of bilayers. Drying under N_2_ stream was performed after every BA-PAA-layer deposition, excluding the very first layer. The resulting ensemble is denoted by BA-PAA-(ChS or PVA#BA-PAA)_**n**_, where BA-PAA represents the identity of the BA-functionalized poly(amido amine) used and **n** represents the number of bilayer. The first BA-PAA layer is regarded as a precursor layer and therefore excluded from the bilayer number count. Typically, the ensemble consists of 10 bilayers with the BA-PAA as the last layer. For every multilayered system, three samples were fabricated in parallel to give estimation for standard deviation. To study the build-up profiles, UV spectra were recorded after each drying step following BA-PAA layer formation. Afterwards, the multilayers were dipped into ChS or PVA solution to continue multilayer build-up.

Multilayers for cell culture were fabricated directly in the wells of poly-D-lysine-coated 96-well plates (PDL-TCPS, Greiner) by alternatingly dispensing deposition (70 μL) and washing (2× 100 μL) solutions under sterile conditions inside the laminar flow hood (LFH). Deposition started with ChS or PVA (2 mg/mL, 30 min for the first layer, 5 min next) as the first layer to a total of 10 bilayers ending with the BA-PAA layer. No intermediate drying steps were applied. At the end of the fabrication the plates were left inside the LFH briefly to dry the films. Coated plates were kept at 4°C and used as soon as possible (typically overnight). These multilayered samples are designated as PDL-(ChS or PVA#BA-PAA)_**10**_ to indicate the presence of a PDL layer as a precursor layer. Compared to multilayers built on quartz and silicon wafer substrates, these systems substitute the first BA-PAA layer with PDL layer inherent to the well plate surface.

### Contact Angle Measurements

Static contact angles (θ) were measured with Milli-Q water (18.2 MΩ∙cm) on a Krüss G10 Contact Angle Measuring Instrument. Five drops of Milli-Q water (approximately 1.5 μL) were measured on five different spots across the surface and averaged to obtain θ. Images were recorded and measured for θ approximately 15 s from the initial contact of the liquid and the surface.

### Atomic Force Microscopy

Multilayers built on silicon wafer substrates were used for AFM microscopy. Imaging was carried out in the middle part of the films (to avoid edge effects/defects) at three different 20 × 20 μm scan areas at 512 × 512 pixels. Images were taken and analyzed using Nanoscope software version 7.30. Height data was flattened using first or second order fitting and root mean square roughness (RMS) was calculated over the scan area. Thickness data was obtained by first scratching the surface using a syringe needle and then scanning over the scratched area to measure the difference in height between the base substrate (*i.e.*, the bottom of the scratch) and the surface of the film. Thicknesses were then measured as the average of one ‘depth’ measurement and three ‘section’ measurements across the scratch.

### Stability Profiles Under Physiological Conditions

Stability profiles of the six different multilayers in PBS buffer pH 7.4 at 37°C was investigated by dipping the thin films formed on quartz slides in 2 mL of PBS buffer pH 7.4 solution and incubating them in a water bath with temperature set to 37°C. From time to time, the samples were removed, briefly dipped in a large amount of Milli-Q water, dried under N_2_ stream and measured by UV–vis spectrophotometer.

### Multilayer Degradation Under Reducing Conditions

Degradability of the six different multilayers were investigated in a similar way as for the investigation of their respective stability profiles under physiological conditions, though in the presence of 2.5 mM of DTT, 10 mM glutathione, or 0.4 mM glutathione in the incubation media. The solutions containing DTT or glutathione in PBS buffer pH 7.4 were prepared fresh, directly prior to the start of experiment. Due to instability of DTT and glutathione in PBS buffer pH 7.4, no solution of over 3 h old was used.

### Responsiveness to Acidic pH

The responsiveness of the six different multilayers in different acidic pH values was investigated in a similar way as for the investigation into their respective stability profiles under physiological conditions, but with citrate buffered saline (CBS) at pH 4, 5, and 7, instead of PBS at pH 7.4.

### Responsiveness to Various Glucose Concentrations

The responsiveness of the six different multilayers in the presence of glucose was investigated in a similar way as for the investigation of their respective stability profiles under physiological conditions, but in the presence of 10, 20, or 100 mM of glucose in the incubation media.

### COS-7 Cell Viability on Multilayered Films

For cell viability experiments, multilayers were fabricated as described previously. Directly on the multilayer-coated PDL-TCPS wells, COS-7 cells were seeded at a seeding density of 10 000 cells/sample or 31 250 cells/cm^2^ in complete medium with serum and left to proliferate at 37°C in humidified atmosphere with 5% CO_2_.

Cell morphology was recorded after 6 and 48 h of culture. At the end of the 2 day culture period, cells were stained with EthD-1 and Hoechst 33258 followed by subsequent fluorescence imaging. As positive controls, cells were also seeded on non-tissue culture (TC)-treated, polystyrene well plates. Experiments were done in triplicate.

For quantitative metabolic activity comparison between the different multilayers, after 48 h of culture, AlamarBlue (AB) was added at 10 *v/v*% of total medium volume in every well and cells were incubated for another 4 h after which fluorescence intensity of resorufin (*i.e.*, metabolically-reduced resazurin) was recorded using a plate reader. As positive controls, cells were also seeded on non-TC-treated polystyrene well plates. All fluorescence intensities were corrected by subtracting the values with those of their respective no-cell control wells. All experiments were carried out in triplicates.

## Results and Discussion

### Syntheses of the Monomer 4AMPBA and the Copolymers DAB-BA-PAA, ABOL-BA-PAA, and DAB-Bn-PAA

**Scheme 3:**
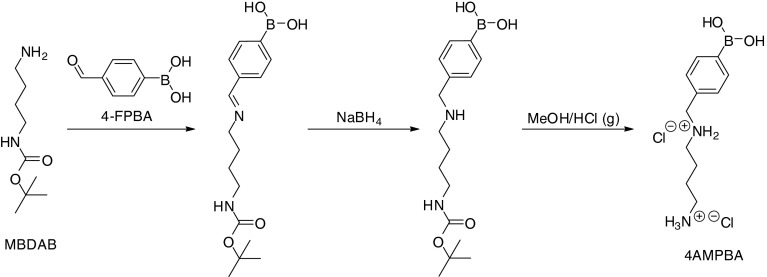
Reaction scheme for the synthesis of monomer 4AMPBA.

Syntheses of the BA-PAAs started with the synthesis of the BA-functional monomer. The monomer 4AMPBA was synthesized as illustrated in Scheme 3. The reaction started with the formation of imine between NBDAB and 4FPBA under nitrogen atmosphere. After 1 day, the light yellow transparent solution turned slightly brown indicating formation of imine which lengthens the double-bond conjugation in the new compound. This newly formed imine bond was reacted with excess of NaBH_4_ upon which the color of the solution turned yellowish again. This color change indicates that the conjugation length in the product is decreased, which is in accordance with the reduction of the imine bond. After isolation under reduced pressure, the product was further purified *via* extraction in DCM/water mixture. The product dissolved more readily in the organic DCM phase while the charged borate salt that was formed as side product dissolved more readily in the aqueous phase. Deprotection by treatment of the product in methanol with HCl gas readily yielded the final product in its HCl salt form. ^1^H-NMR showed the disappearance of the 9-proton singlet in 1.4 ppm which indicated complete deprotection. Following exhaustive lyophilization, the purity of the product was confirmed using a known amount of *N,N*-dimethylformamide as internal standard in ^1^H-NMR sample characterization to be roughly 90–94%.

Syntheses of the three random copolymers DAB-BA-PAA, ABOL-BA-PAA, and DAB-Bn-PAA followed similar procedure as reported previously (16). The ratio of amino monomers DAB/4AMPBA and ABOL/4AMPBA was chosen 50/50 in each BA-PAA to provide a balance between the more polar DAB or ABOL side chain and the more apolar 4AMPBA side chain, which is important to ensure solubility of the copolymer in aqueous solutions. To free the primary amine of 4AMPBA, excess amount of TEA was added to the reaction mixture. Compared to the syntheses of linear PAAs reported previously (16), polymerization solutions of these BA-PAAs reached higher viscosity much faster due to the branching of the benzylic secondary amine. Thus, a portion of the product came out of solution upon reaching high molecular weight. This portion was not readily soluble in water and were not included for further purification through ultrafiltration and lyophilization. Nevertheless, this did not significantly affect polymerization yield, except for the control polymer DAB-Bn-PAA where the majority of product came out of solution during BOC-deprotection and was lost, likely due to the higher hydrophobicity of the non-boronated polymer.

**Fig. 1:**
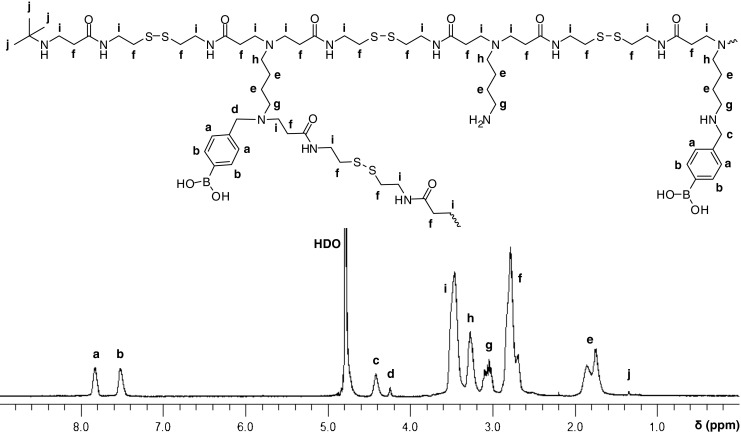
^1^H-NMR spectrum and structural elucidation of DAB-BA-PAA.

The ^1^H-NMR spectrum and structural elucidation of DAB-BA-PAA are shown in Fig. 1. The other two copolymers provide comparable spectra and their structural elucidations are carried out similarly. All of the three spectra show no trace of unreacted acrylamide groups which are known to be toxic. The degree of BA or Bn-functionalization could be estimated from the integration values of the aromatic protons in relation to the protons of cystamine or butylene moiety. Presence of two singlets at 4.42 and 4.25 ppm indicates branching in the secondary amine of the 4AMPBA monomer. Reacted secondary amine of the 4AMPBA monomer would have the methylene protons chemical shift shifted upfield (Fig. 1d) from that of the unreacted 4AMPBA secondary amine (Fig. 1c). The ratio of integration values of the more upfield singlet (Fig.1d) as compared to the total (Fig. 1c, d) integration values of the two singlets indicates that ~19% of the incorporated 4AMPBA reacted further through its secondary amine, translating to a total of ~10% branching.

**Scheme 4:**
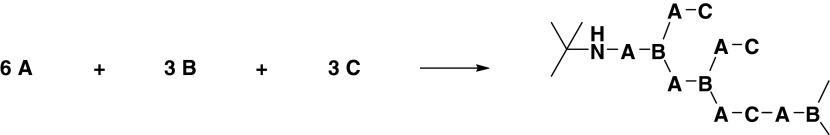
The schematic stoichiometric reaction with *A* = difunctional CBA, *B* = difunctional BA or Bn containing amine, and *C* = monofunctional amine.

Number average molecular weight (M_n_) can be estimated *via* the *t*Bu end group (Fig. 1j). Considering the 1:1.5 CBA:reactive amine ratio, and assuming only one *t*Bu end group is present per polymer chain to react with a single unreacted acrylamide end in the branched polymer chain (Scheme 4), the number of repeating unit is ~27, translating to M_n_ of 11.5 kg/mol. Table I summarizes the ^1^H-NMR elucidation for the three random branched copolymers synthesized. Attempts to obtain molecular weight (MW) and PDI *via* GPC did not provide satisfactory result, due to the BA interaction with the polyol-based stationary column.

**Table I:** Summary of ^1^H-NMR Spectra Elucidation (%BA or Bn Functionalization, Degree of Branching, Number of Repeat Unit, and M_n_) of the Branched PAA Copolymers

PAA name	%BA or Bn		% Branching	No of repeat unit	M_n_ (kg/mol)
	%repeat unit	%branched			
DAB-BA-PAA	53.5	19.1	10.2	27.2	11.5
ABOL-BA-PAA	52.5	59.3	31.1	31.9	13.4
DAB-Bn-PAA	60.0	21.4	12.8	23.4	9.5

**Scheme 5:**
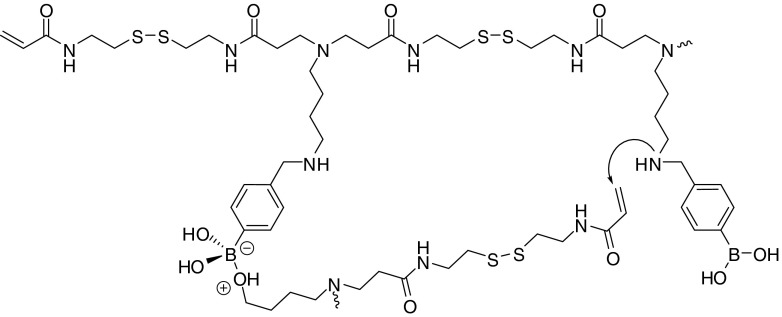
Schematic illustration of possible interaction between primary alcohol and boronic acid side groups of ABOL-BA-PAA, thereby increasing affinity between reactants.

As shown in Table I, both the molecular weight and the degree of branching are relatively low in comparison with some reports on the one-pot-synthesized hyperbranched polymers (37,38) but are in agreement with a previous report utilizing the same CBA, ABOL and similar reaction procedure (29). Although this previous report concluded no significant difference in the branched vs linear PAA, we note significant increase in molecular weight of BA-containing PAAs reported in this study in comparison with previously reported linear BA-containing PAA (16). The previously reported linear BA-PAA was synthesized at 50°C. The reactivity of the secondary amine in 4AMPBA and BnDAB was most likely enhanced by the increase in reaction temperature to 70°C. The increase in reactivity is not significant as indicated by the relatively low degree of branching. In addition to the moderate increase in reaction temperature, branching was most likely limited by polymer solubility in the polymerization solvent (a high molecular weight insoluble portion was filtered off), and steric hindrance. It is interesting to note that ABOL-BA-PAA displayed a higher degree of branching as compared to DAB-BA-PAA. The interaction of the alcohol side chains with boronic acid possibly increases affinity between reactants (Scheme 5). Moreover, ABOL-BA-PAA may be more soluble than BOC-protected DAB-BA-PAA during polymerization, making it easier for the former to reach higher degree of polymerization.

In relation to multilayer build-up, it is hypothesized that the presence of branching in this polymer is beneficial in providing more multivalent interactions to stabilize the construct. Since the deposition involves mainly the interaction of the polymer with a surface, the effect of minor differences in MW is expected to be less prominent than the chemical features of the three polymers. Herein the structure-function relationships are described mainly in terms of BA and Bn functionality in the polymers, their interactions with ChS and PVA, and the possible boronate ester formation.

### Multilayered Thin Film Build-Up: Effects of Molecular Weight of PVA

**Fig. 2:**
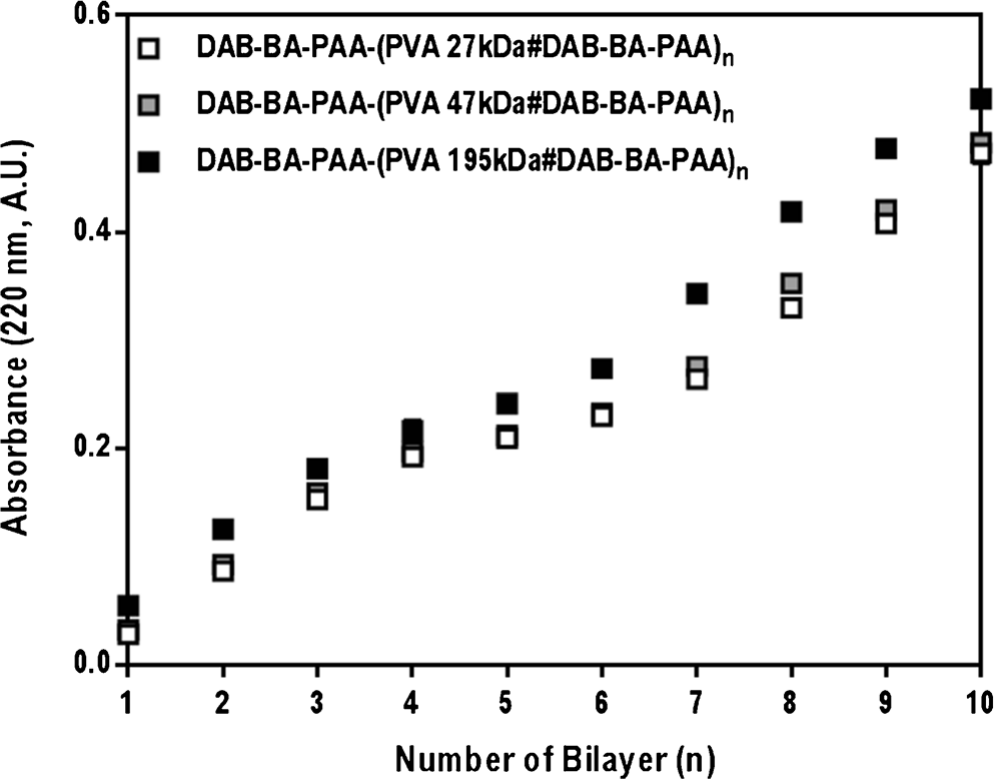
Build-up profiles of DAB-BA-PAA polymer with different molecular weight PVA.

PVA is commercially available in various molecular weights (MW) and therefore provides an excellent means to study the effect of PVA molecular weight on multilayer build-up. With increasing PVA MW, the relative diol concentration in the deposition solution increases while the BA concentration stays constant. The three different molecular weights of PVA chosen are 27, 47, and 195 kDa. Multilayer formation was carried out as mentioned in the “Materials and Methods” section. Build-up profiles of the DAB-BA-PAA polymer with three different PVA molecular weights are shown in Fig. 2. Absorbance values stated are those at the maximum wavelength of absorption through UV spectroscopy as explained further in the next section.

Figure 2 shows that regardless of the molecular weight of PVA used, the build-up profile remains linear and to similar extents. Increasing diol concentration in the PVA deposition solution does not lead to pronounced increase in BA-PAA deposition. This is very much in contrast to the findings on PVA-based films utilizing weak hydrogen bonding as the only driving force (39) and serves as an indication that the boronic ester formation plays a major role in the build-up of the multilayered system of DAB-BA-PAA and PVA. As the diol concentration is very much in excess to the BA concentration, not much increase in BAPAA deposition is observed upon increasing the molecular weight of the PVA as no additional boronic ester formation is possible. Similarly, multilayer stability under physiological conditions was also found to not be affected by the difference in PVA molecular weight, although there is a notable increase in surface roughness as the PVA MW increases due to folding needed for longer chains to be deposited on the surface (data not shown). The lowest PVA MW (*i.e.*, 27 kDa) is sufficient in facilitating optimal deposition and build-up of the BA-containing polymer and keeping the construct stable under physiological conditions. This PVA MW is selected for the following subsequent studies.

### Multilayered Thin Film Build-Up: ChS *versus* PVA

**Fig. 3:**
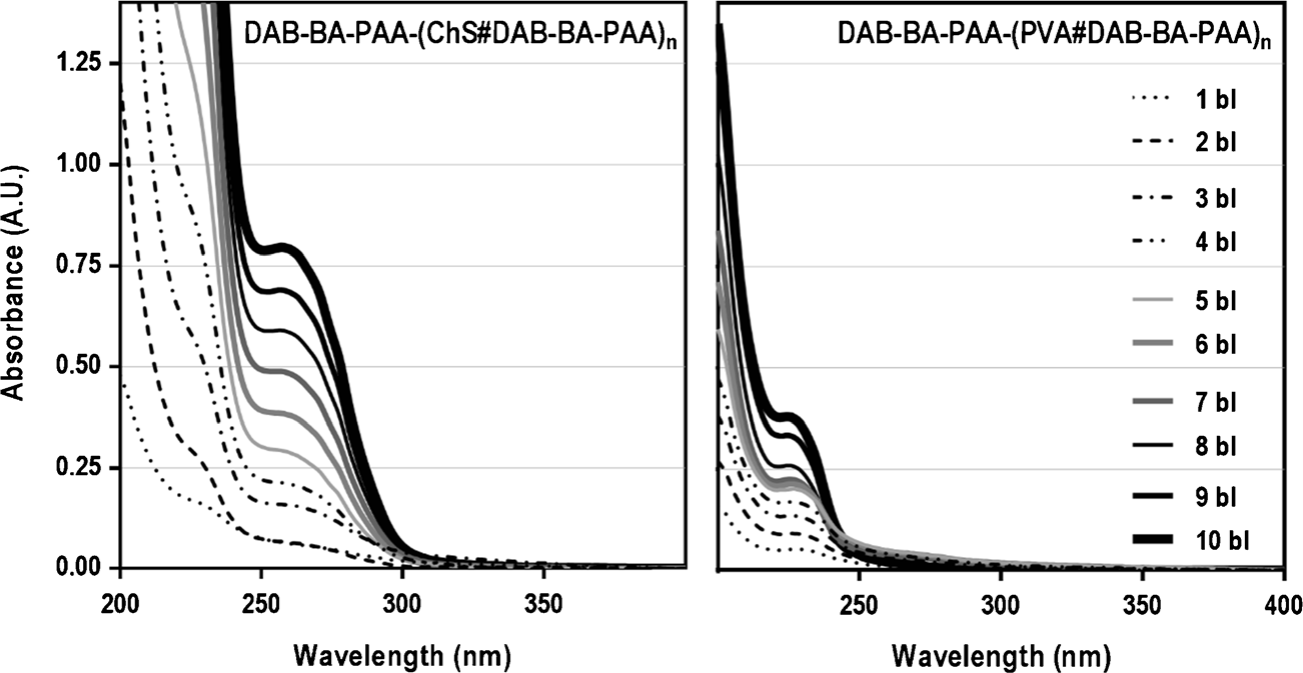
UV spectra of DAB-BA-PAA-(ChS#DAB-BA-PAA) and DAB-BA-PAA-(PVA#DAB-BA-PAA) at various bilayer numbers.

As indicated in the previous section, the study of build-up profiles and the relative incremental increase in material deposition was carried out through the UV spectra of the multilayers in the dry state which were recorded after every deposition cycle, starting from 1 bl (with BA-PAA layer on top). The UV spectra of the different multilayers were similar with respect to whether DAB-BA-PAA, ABOL-BA-PAA, or DAB-Bn-PAA was employed as the BA- or Bn-functionalized polymer. Therefore, as a representative example, the UV spectra of DAB-BA-PAA-based systems at increasing bilayer numbers are displayed in Fig. 3. Depending on whether ChS or PVA was incorporated, the UV spectra had maxima at 258 and 227 nm, respectively. On the ChS system, the maximum at 227 nm appears as a shoulder with much higher absorption that exceeds the ideal absorption of ≤ 1 A.U. for maintained linearity at higher bilayer numbers. Therefore the more reliable maximum at 258 nm is used for further study on the ChS system.

**Fig. 4:**
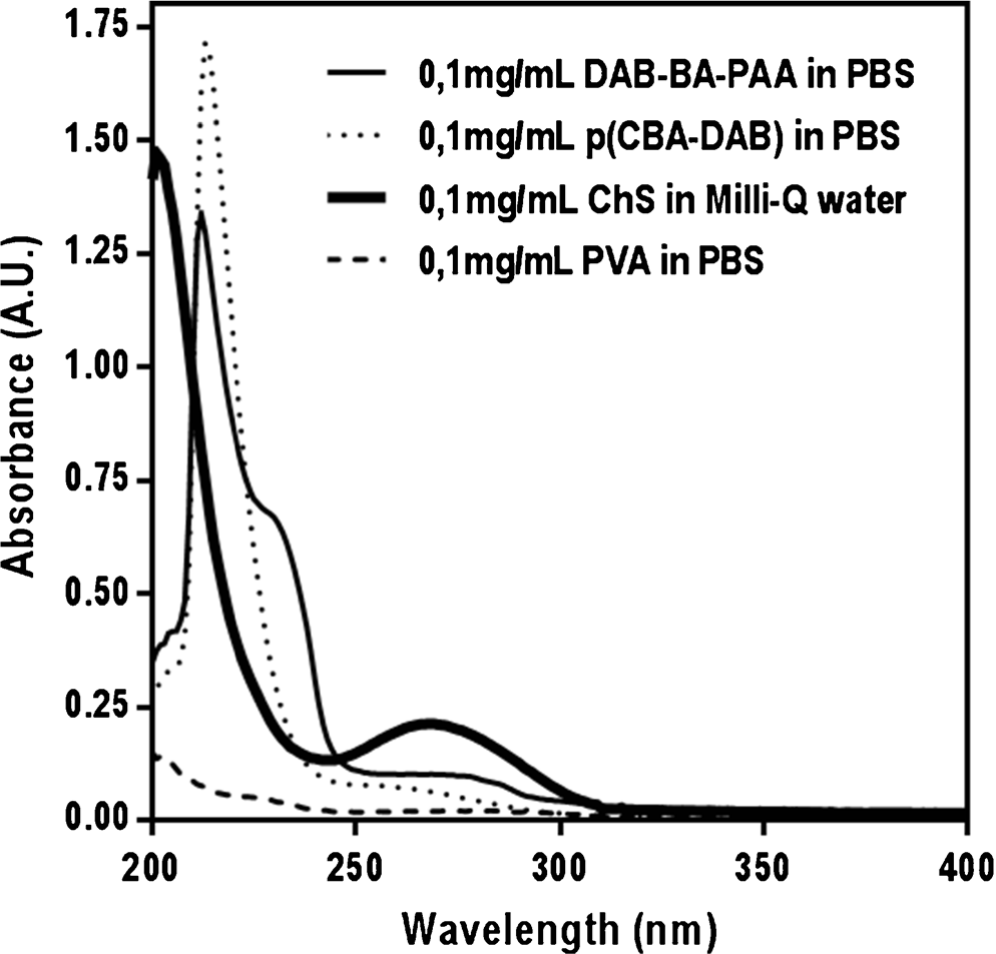
Solution state UV spectra of relevant multilayer components.

To understand the origin of the observed absorption maxima, the UV spectra of the solutions of relevant multilayer components are shown in Fig. 4. From this figure, the maximum at 227 nm can be attributed to the absorption of the phenyl moiety of DAB-BA-PAA, (ABOL-BA-PAA and DAB-Bn-PAA, data not shown), which is also confirmed by the spectrum of the p(CBA-DAB) homopolymer solution where such a signal is not observed. The maximum at 258 nm is attributed to the ChS which shows a distinct peak with notably relatively higher extinction coefficient than the absorption of other components at the same wavelength and concentration.

**Fig. 5:**
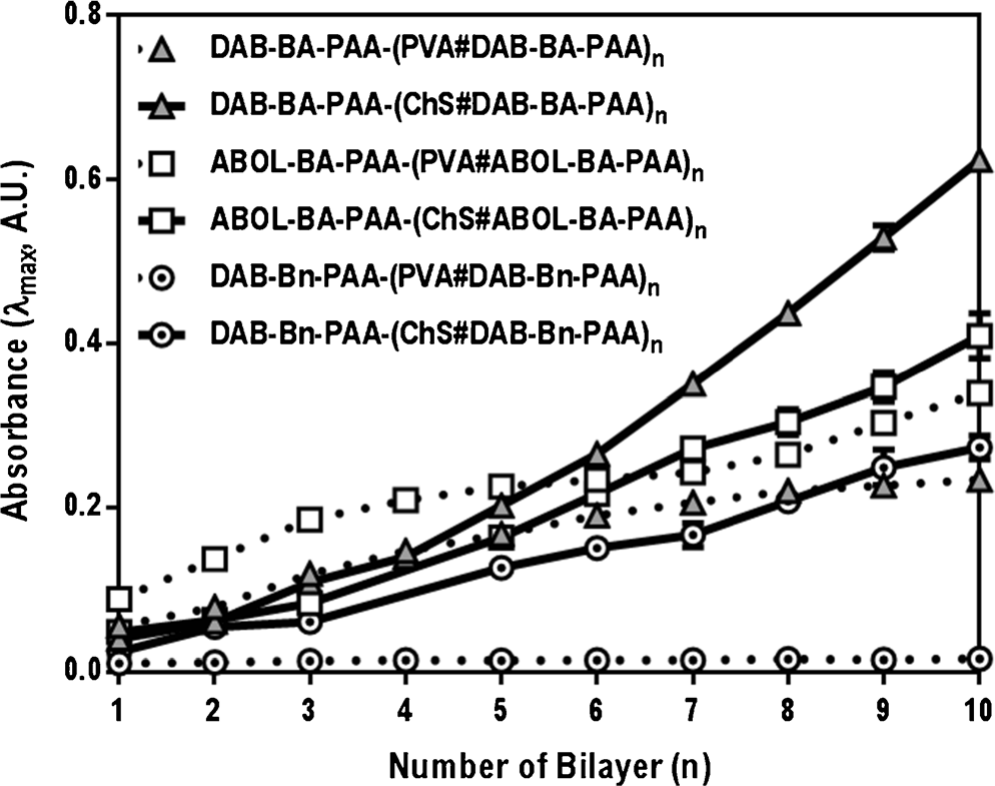
Build-up profiles of DAB-BA-PAA-(PVA#DAB-BA-PAA), DAB-BA-PAA-(ChS#DAB-BA-PAA), ABOL-BA-PAA-(PVA#ABOL-BA-PAA), and ABOL-BA-PAA-(ChS#ABOL-BA-PAA) multilayers. Absorbance values shown are those at 227 nm for PVA-based films and 258 nm for ChS-based films.

Based on the respective absorption maxima, build-up profiles can be plotted for the multilayers. All of the BA-based systems exhibit relatively linear build-up profiles as shown in Fig. 5. The control polymer DAB-Bn-PAA, however, provides a remarkable finding. For this polymer which does not contain a BA moiety, multilayer build-up is positive when paired with ChS, but negative when paired with PVA. This phenomenon indicates that the positive multilayer build-up of PVA-based films in this study depends substantially on the boronate ester formation. This finding also implies that multilayer build-up with ChS may still progress without boronate ester formation.

**Fig. 6:**
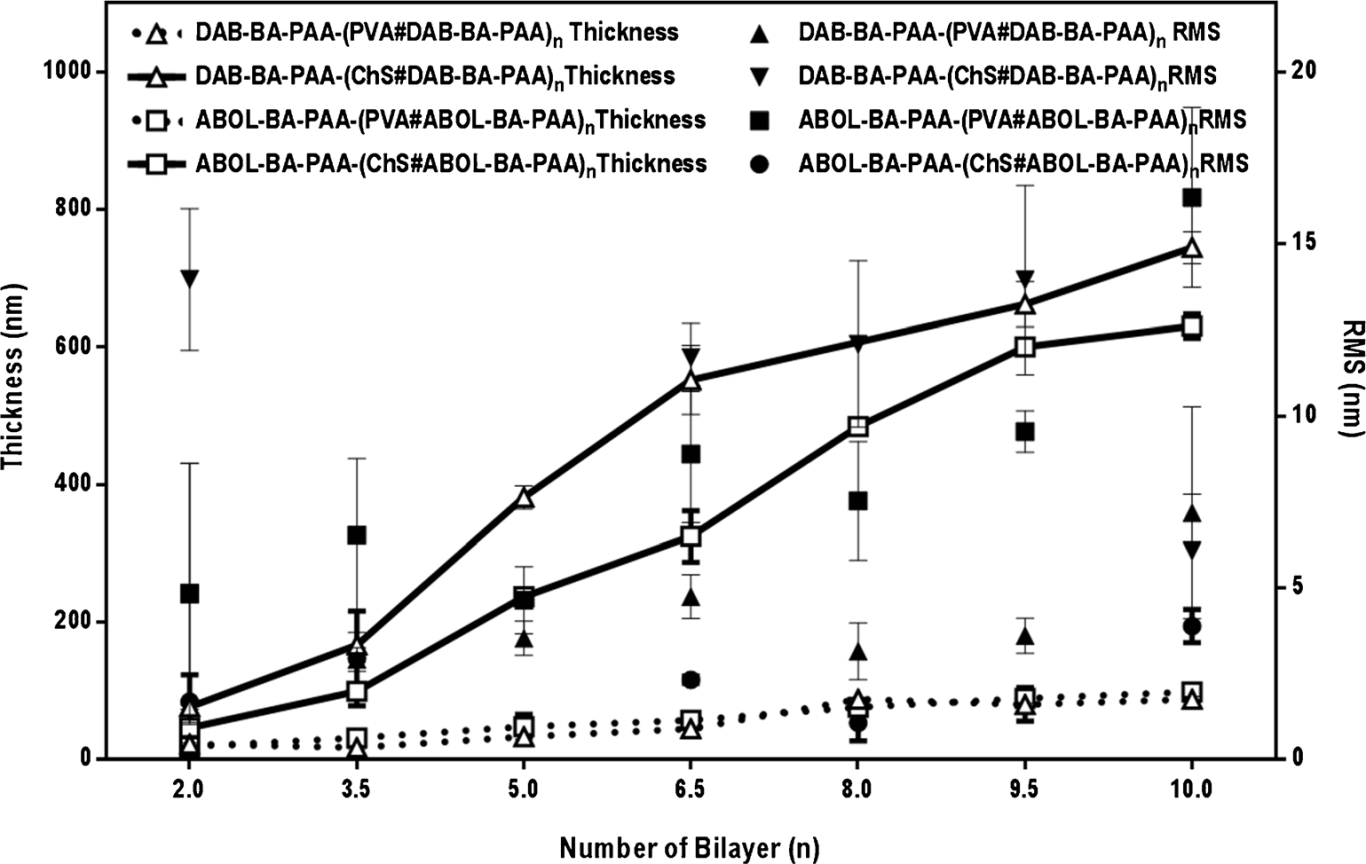
Build-up profiles of the multilayered systems based on AFM. Surface roughness (*RMS*, nm) and thickness (*nm*) at different bilayer numbers are shown at the two ordinate axes. Every data point is an average of at least three measurements made on arbitrary positions of the same multilayer sample.

To complement the UV-spectrophotometry data, thickness data was also obtained through AFM scratch tests, *i.e.*, a scratch was made using a small syringe needle across the surface of the film which was further imaged using AFM. Thickness and surface roughness data obtained for the four BA-based multilayers on various bilayer numbers are plotted in Fig. 6.

Figure 6 agrees well with the UV spectrophotometry data displayed in Fig. 5 in terms of relative build-up profiles of the different systems. However, it is remarkable to note the tremendous difference in thickness between PVA- and ChS-based films, which is not apparent from the UV data due to the difference in λ_max_ (and subsequently extinction coefficient) used for the different systems. At the same 10 bilayers, ChS-based systems were at least 600 nm thick while PVA-based systems were much thinner at ~100 nm. The thick nature of the ChS-based film was also observed qualitatively by eye through the increased turbidity, especially at higher bilayer numbers. In a computational study, Rodríguez-Carvajal *et al.* reported that chondroitin 4-sulfate (the ChS variant used in this study) particularly assumes a rigid helical conformation (40), a feature that also contributes to ChS properties as bone extracellular matrix. Moreover, unlike the BA-PAAs, ChS is a strong polyelectrolyte. Taking into account the findings on the effect of charge density on multilayer build-up (41,42), it is even less likely that ChS would take up more globular conformation due to charge repulsion. Therefore, the very thick nature of the (ChS#BAPAAs) multilayers can only be attributed to the increased amount of deposited ChS. As a strong polyelectrolyte, ChS readily binds counter-ions. Especially for chondroitin 4-sulfate, it is reported that small counter-ion binding sites are energetically more favorable (40). This may facilitate deposition of more ChS chains, effectively increasing the multilayer thickness.

Upon closer examination of the build-up profiles (Figs. 5 and 6), ChS-based films (solid lines) notably display more linear build-up profiles, while for PVA-based films (dotted lines), multilayer growth slows down after approximately 5 bilayers. This change in build-up profile upon 5 bilayer formation (also observed in Fig. 2) may be explained as an effect of the underlying charged-substrate, which may be particularly prominent in a system where the driving force for multilayer build-up is not electrostatic interactions (43–45). The lower thickness of the PVA (in contrast to ChS) system may also contribute to increase the effect.

Another interesting observation can be made from Fig. 5 on the relation of the extent of material depositions to the two different polymers. For ChS-based films, DAB-BA-PAA promotes higher deposition than ABOL-BA-PAA, while for PVA-based films, the contrary is observed. Since the two polymers possess similar degrees of BA functionalization (Table I), this finding may be explained by the difference in the type of side group (*i.e.*, DAB vs ABOL) of the two polymers, and their effect on overall charge densities of the polymers and interactions with the other multilayer components. DAB-BA-PAA with protonated primary amine side groups has a higher cationic charge density and consequently provide stronger electrostatic interactions with the negatively charged ChS than ABOL-BA-PAA with primary alcohol side groups. Thus, DAB-BA-PAA favors interaction with negatively-charged ChS, while ABOL-BA-PAA favors interaction with PVA, possibly because the hydroxybutyl chains can better accommodate in the PVA layer than the charged DAB chains. These results indicate that possible stronger interactions lead to higher incremental increases of the paired multilayer components. However, this is in contrast to the widely accepted findings that less, but sufficiently-charged polyelectrolytes give rise to increased deposition (42,46). This finding is most likely explained by the presence of more hydrophobic BA moieties in the random branched polymer structure. As previously reported, *ortho*-substituted linear ABOL-BA-PAA spontaneously form nanosized particles in solution, which was attributed to interactions of BA moieties with the primary alcohol side groups in the proximity (16). The same observation was made for the branched ABOL-BA-PAA in this study. Upon dissolution of the branched ABOL-BA-PAA in glucose-free HEPES buffer pH 7.4 at 0.6 mg/mL, dynamic light scattering (DLS) revealed the presence of particles 94 nm in size with PDI of 0.3. The same phenomenon was observed for the branched DAB-BA-PAA, albeit with much higher PDI of 0.6, indicating a less homogenous particle size distribution. Furthermore, the ABOL-BA-PAA particles have noticeably more positive zeta potential as compared with DAB-BA-PAA (up to +40 and +4 mV, respectively). This indicates a significant difference in the conformation of the two copolymers in solution, which may also play a role in the way they interact with a surface. Most notably, the apparently higher positive charge of ABOL-BA-PAA in solution may lead to stronger interaction with ChS, which further leads to lower incremental increase during layer build-up, as compared with DAB-BA-PAA. On PVA-based films, the higher deposition provided by ABOL-BA-PAA may be due to the ability of primary alcohol groups to facilitate boronate ester formation better than (charged) primary amine groups (39).

### Contact Angle Measurements

**Fig. 7:**
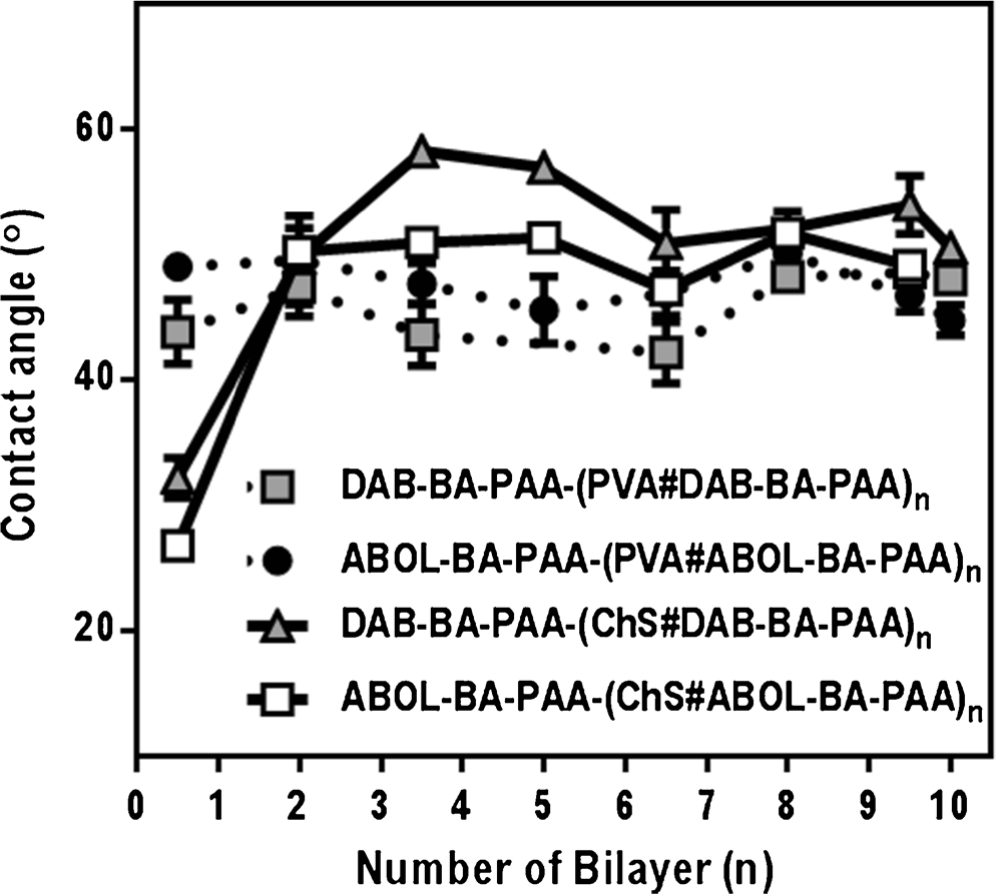
Static water contact angles of multilayers at different numbers of bilayers.

To examine whether different static water contact angles can provide information on the identity of the topmost layer and the hydrophobicity of the surfaces in general, multilayered systems with different bilayer numbers were measured *via* the sessile drop method (Fig. 7). Figure 7 shows that at least for the first 2 cycles of deposition of ChS-based multilayers, layer coverage is incomplete and the effect of the bare hydrophilic silicon wafer substrate can still be observed through the lower contact angle values. For the PVA-based multilayers, however, complete surface coverage can be observed as early as the 0.5 bilayer (*i.e.*, film with first precursor polymer layer and the first PVA layer), probably because of the more specific interactions PVA has with the BA-PAA layer.

At higher bilayer numbers, the contact angle values do not show a marked difference when the top most layer was either the BA-PAA layer, or the ChS or PVA layer. Serizawa *et al.* reported the average contact angle values of 45° for PVA top-most layers for their LbL-assembled multilayers of PVA with poly(methyl methacrylate) (PMMA) (47), which is relatively similar to the observed value shown in Fig. 7. Literature reports on contact angles of ChS film mention values between 70° and 80° (48,49) depending on prior treatment and the nature of the underlying surfaces.

Instead of the static contact angles, advancing and receding contact angles may provide more accurate information on surface profile and hydrophobicity. Moreover, the N_2_ drying step employed during film formation may not completely dry the system from trapped water molecules, especially considering the hygroscopic nature of all of the layer components, and therefore may substantially affect the static contact angle values observed. Nevertheless, the obtained data provides information on the onset of complete surface coverage which has to be taken into account for subsequent experiments. The data may also be more relevant for the intended applications where the films are stored at ambient humidity, at room temperature or 4°C with no notable differences in wettability. In agreement with the contact angle data, the 10-bilayered ChS-based films were always less easily wetted by aqueous solutions (such as cell culture medium) than the respective PVA-based films, regardless of the presence of a BA-PAA topmost layer.

### Atomic Force Microscopy Surface Profiles

**Fig. 8:**
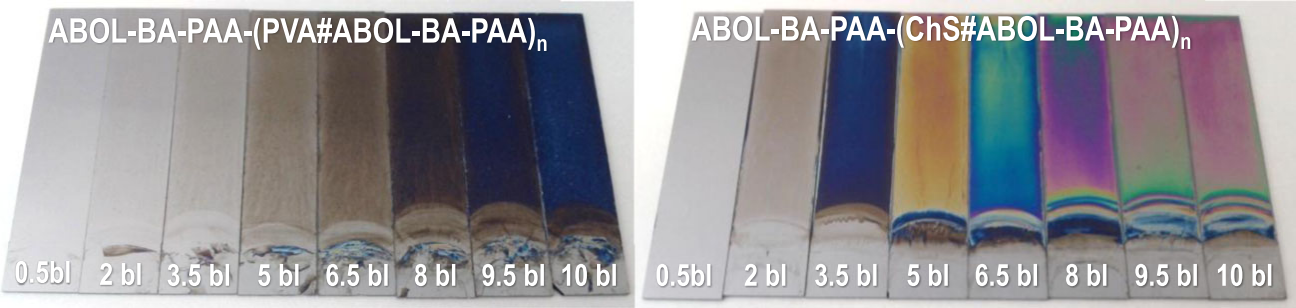
Digital photograph of ABOL-BA-PAA-(PVA#ABOL-BA-PAA) and ABOL-BA-PAA-(ChS#ABOL-BA-PAA) built on silicon wafer at various bilayer numbers.

In addition to providing thickness information, AFM was also used to gain insights into the possible differences in surface profiles of the different films, and at different bilayer numbers. A digital photograph of the complete set of ABOL-BA-PAA-based samples, both with PVA and ChS at various bilayer numbers is shown in Fig. 8. DAB-BA-PAA-based films look similar, but with less homogeneous surfaces and increased turbidity. Figure 8 shows the typical thin-film interference color pattern observed during build-up on silicon wafers of various multilayers reported in this thesis. Notably, films of ~100 nm thick show deep blue interference color as shown for ABOL-BA-PAA-(PVA#ABOL-BA-PAA)_**10**_ in Fig. 8. In agreement with the thickness data, ABOL-BA-PAA-(ChS#ABOL-BA-PAA) reaches the same thickness much earlier at 3.5 bl and shows the same deep blue color.

Similar to the contact angle data, the four multilayered systems do not show alternating surface profiles as observed from AFM. Typical surface profiles of the four different systems shown using the same height scale of ±40 nm are shown in Table II. All of the PVA-based films show sharp height features indicating high surface roughness. As shown in Fig. 6, PVA-based films have roughness up to 16 nm at 9.5 and 10 bl. This is typically much higher compared with ChS-based films, especially considering the much thinner nature of the PVA-based films. ChS-based films show very different surface profiles depending on whether it is paired with DAB-BA-PAA or ABOL-BA-PAA. (ChS#DAB-BA-PAA) films are much rougher at all stages of the build-up, with roughness up to 15 nm. Interestingly roughness is enhanced with ChS as the topmost layer. In comparison, (ChS#ABOL-BA-PAA) films are smooth at all stages of the build-up, with the highest roughness obtained at less than 5 nm at the highest bilayer number. This difference in roughness of the two ChS-based films were also observed visually during multilayer build-up.

**Table II:**
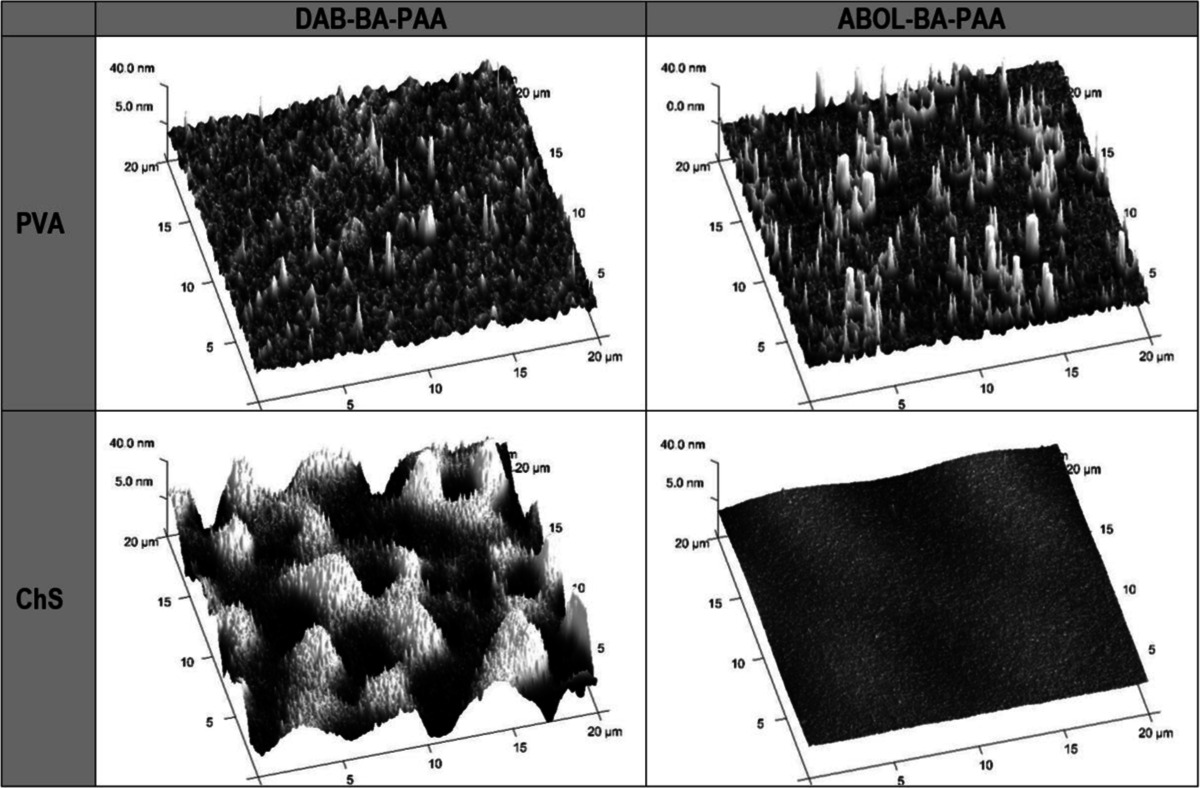
Typical Surface Profiles of the Four Different Multilayers Based on AFM

Images were recorded on 9.5-bilayered films, with PVA or ChS topmost layer. All images were scanned over 20 × 20 μm areas and height scales were maintained at ±40 nm

### Stability Profiles Under Physiological and Reducing Conditions

Investigating multilayered film behavior under physiological conditions may serve to assess their stability in the presence of physiological salt concentration, pH, and temperature. The stability profile may be used to tune the film’s behavior, for example, in releasing loaded drugs. Further, both DAB-BA-PAA and ABOL-BA-PAA contain disulfide bonds in the main chain to render them biodegradable under reducing conditions. This could especially be important when the BA-PAAs are taken up by cells, since it has already been shown that polyplexes of these polymers rapidly degrade in the reducing cytosol due to the presence of a relatively high concentration of glutathione (~5–10 mM) (50). Moreover, in biological medium, extracellular reductases may contribute to film degradation due to reductive cleavage of the disulfide bonds (51). To investigate the stability profiles of the four different films under physiological and reducing conditions, the films were incubated in PBS solutions pH 7.4 at 37°C in the presence or absence of either glutathione (0.4 and 10 mM) or DTT (2.5 mM). From time to time, the films were retrieved, dried under a gentle N_2_ stream, and measured for their absorbance values at the respective λ_max_.

**Fig. 9:**
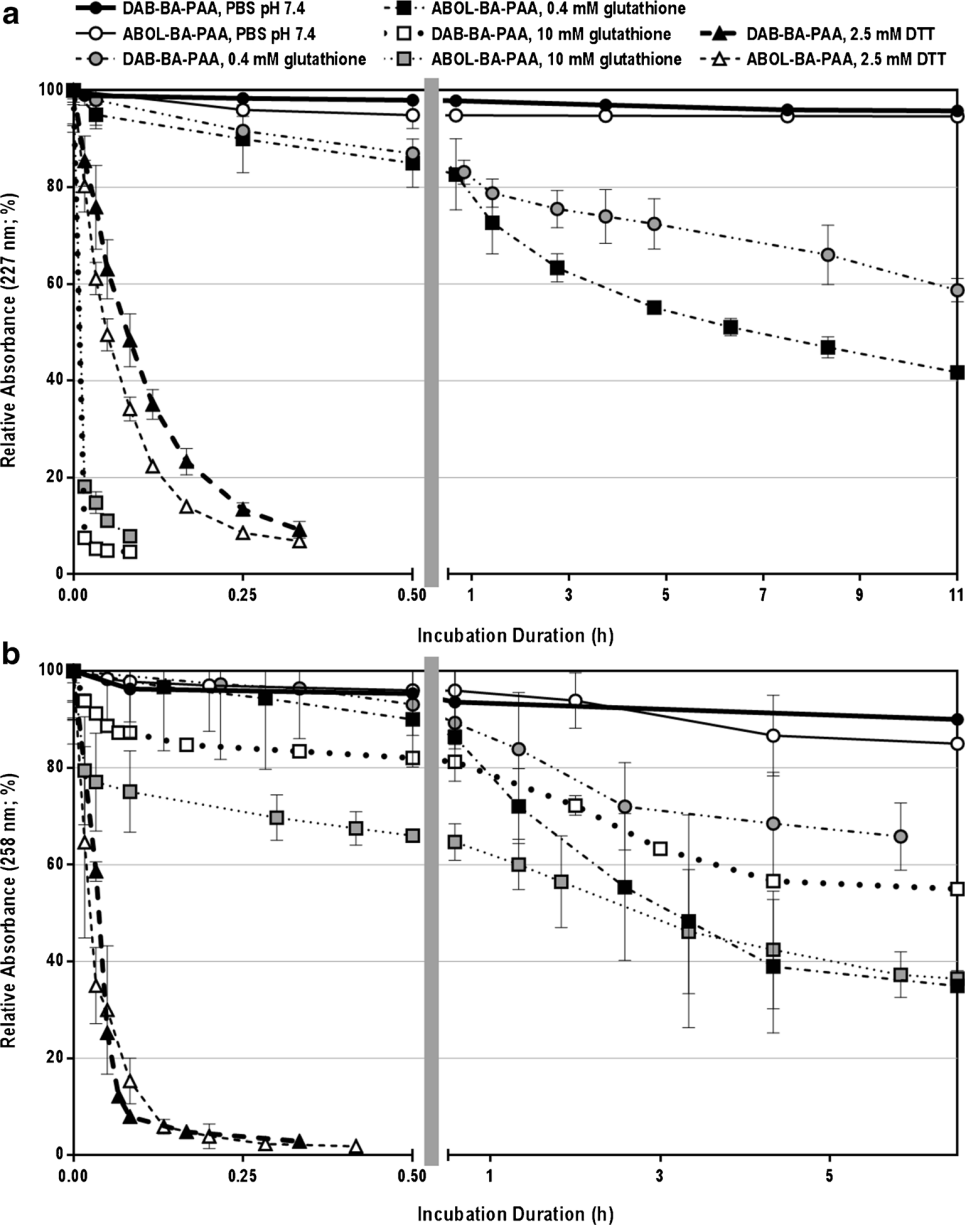
Stability profiles of: (**a**) PVA- and (**b**) ChS-based multilayers under physiological conditions (PBS pH 7.4 at 37°C) and in the presence of glutathione (0.4 and 10 mM) or DTT (2.5 mM). The left part of the abscissa shows the trend of fast decrease (burst release) within 0.5 h (30 min) in the initial phase of the incubation while the right part shows the much slower decrease throughout the rest of the long incubation duration.

Figure 9 shows the decrease in relative absorbance values in time, *i.e.*, the ratio of absorbance value against the respective value at time zero. Figure 9a and b show the results for PVA- and ChS-based systems, respectively. Throughout the incubation time, the four multilayers remain stable under physiological conditions, but are readily degraded in the presence of reducing agents. Higher concentrations of reducing agent degraded the films faster as shown by the use of 0.4 and 10 mM glutathione. It is interesting to note that at 2.5 mM, DTT shows a higher reducing rate than 10 mM glutathione for ChS-based films, but lower for PVA-based films. Glutathione has an extra negative charge due to the presence of glutamic acid in this molecule. It is therefore more negative than DTT and thus may experience more electrostatic repulsion in ChS films. This does not play a role in PVA films. Moreover, from both the UV spectroscopy and thickness characterizations, it is likely that in addition to having much higher thickness, the ChS-based films also contain higher portion of ChS in comparison to the degradable BA-PAAs and therefore, diffusion of reducing agents into the inner part of the film may be slightly impeded relative to the thinner PVA multilayers. This may further explain the high standard deviation observed for the ChS systems.

### Stability Profiles at Acidic pH

**Fig. 10:**
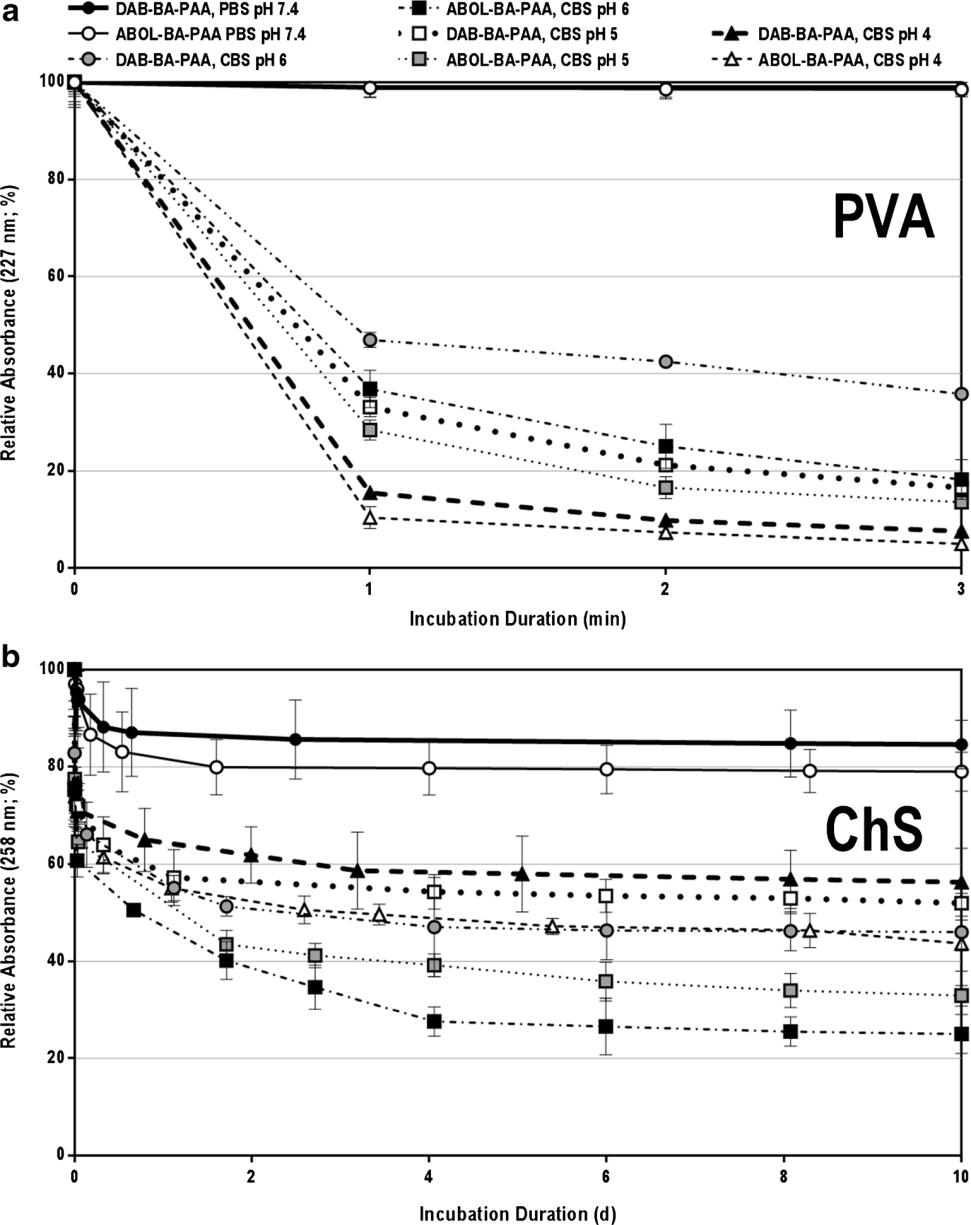
Stability profiles of: (**a**) PVA- and (**b**) ChS-based multilayers under physiological conditions (PBS pH 7.4 at 37°C) and in CBS pH 6, 5, and 4.

As indicated in Scheme 1, the boronate ester formation is sensitive to pH. It is reported that the boronate ester is more stable in tetragonal state (basic conditions) than in the trigonal state (4,52). Thus, boronate ester formation is not favored at acidic pH where the trigonal boronic acid dominates. To investigate the responsiveness of the four different multilayers to acidic pH, the different films were incubated in citrate buffered saline (CBS) at pH 4, 5, and 6 at 37°C. From time to time, the solutions were refreshed, the films were dried, and the relative absorbance is measured as the ratio of the absorbance at a specific time to that at time zero. Figure 10a and b show the stability profiles of PVA- and ChS-based films, respectively. The PVA-based films are notably very responsive to acidic conditions, with faster film dissolution at lower acidic pH. Notably, complete dissolution can be achieved after only 3 min of incubation at pH 4. The high responsiveness of the PVA systems may further indicate the strong BA-diol interaction within the multilayered construct. Low pH values diminish possible ester formation that hold the films together. The very rapid kinetics also signify that there is likely only limited significance of other types of interactions present that could be strong enough to prevent the multilayer from dissolution at low pH.

On the other hand, the ChS systems show much slower dissolution with fast initial response only during the first 1 h. Very interestingly, the disassembly rate is faster at elevated pH as opposed to lower pH. For instance, for the (ChS#ABOL-BA-PAA) system, 70% of disassembly is reached after 4 days of incubation at pH 6 while only ~40% is reached under the same conditions at pH 4. The films are, however, relatively stable under physiological pH, with only ~20% disassembly after 2 days of incubation. This trend may further confirm the low tendency of boronate ester formation in ChS-based multilayers. The phenomenon may be explained by the presence of carboxylic acid groups in ChS. In contrast to the sulfate groups, which are negatively charged at all pH values investigated in this study, the carboxylic acid groups having pKa in the range of ~3-5 may undergo drastic change in ionization state in the acidic pH range of 4–6. As such, at pH 4, ChS is much less negatively-charged than at pH 6. On the other hand, more of the amine of the BA-PAAs are protonated at pH 4 than at pH 6. The overall effect may be the relative higher stability of the system at pH 4 than at pH 6. Under the physiological pH of 7.4, the amines of BA-PAAs are even less positively-charged, but the ChS is fully ionized, leading to a stable system. This stability may be more enhanced by the fact that the films were immersed in the same solvent in which build-up took place, causing less effect of osmotic ion diffusion and destabilization.

### Stability Profiles in Various Glucose Concentrations

**Fig. 11:**
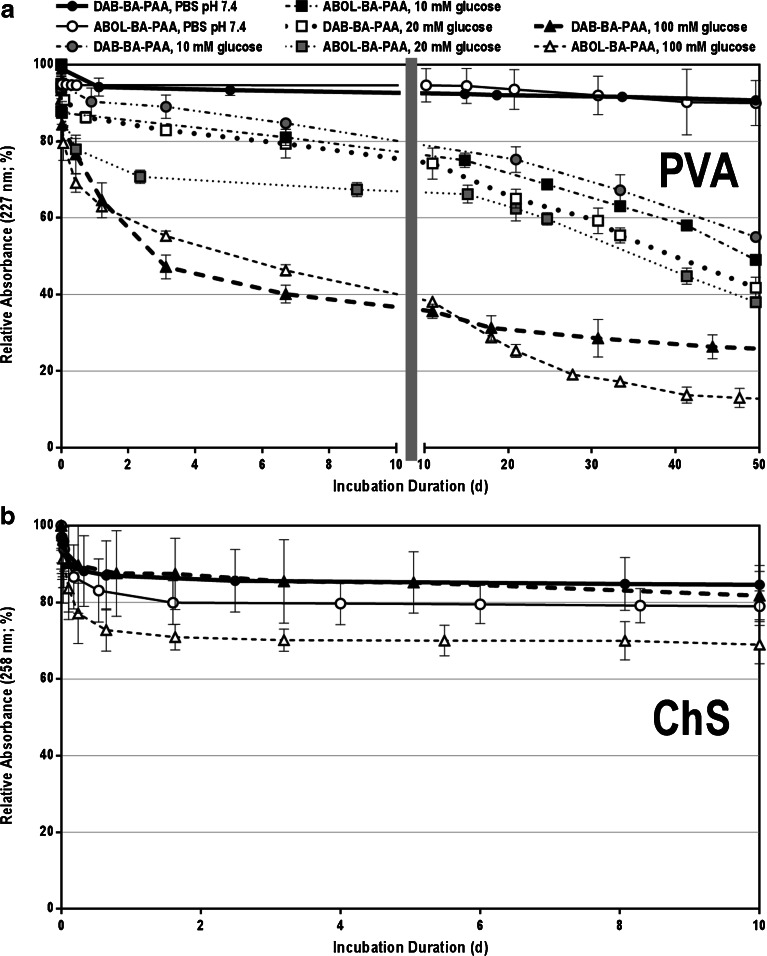
Stability profiles of: (**a**) PVA- and (**b**) ChS-based multilayers under physiological conditions (PBS pH 7.4 at 37°C) and in the presence of 10, 20, and 100 mM glucose. For ChS systems, only the 100 mM glucose data is shown for clarity.

As indicated in Scheme 1, boronate ester formation may be affected by the presence of other diol moieties, such as glucose, through competitive binding. To investigate whether the multilayered systems possess glucose-responsiveness, the multilayers were incubated at various glucose concentrations under physiological conditions. Figure 11a and b show the stability profiles of PVA and ChS systems, respectively. From this figure it can be observed that the PVA systems are more responsive to glucose compared to the ChS system, especially apparent at the highest glucose concentration (100 mM). (ChS#DAB-BA-PAA) films show similar profiles with and without the presence of 100 mM glucose, while (ChS#ABOL-BA-PAA) systems seem to be only slightly responsive to glucose at the same concentration. At lower concentration, the ChS-based films were progressively more stable or identical to their profiles under physiological conditions (data not shown). The low responsiveness of the ChS system to elevated glucose concentrations, despite the long incubation duration, may be due to the more electrostatic nature of the interactions, possibly further enhanced by the thickness of the film limiting diffusion of glucose. As this may indicate that the majority of BA-moieties in the multilayered film are free of ester formation with ChS, this system may be utilized as a depot for drugs which have affinity to the BA-moiety, thereby providing more controlled or triggered release. This possibility is currently under investigation.

The PVA-based films on the other hand, show a clear response to glucose, although with slow kinetics. (PVA#ABOL-BA-PAA) notably reached the same 70% relative absorbance values as (ChS#ABOL-BA-PAA), but at a glucose concentration of only 20 mM, instead of 100 mM for the latter. At 100 mM glucose concentration, (PVA#ABOL-BA-PAA) reaches 40% degree of disassembly following 2 days of incubation. The rate decreases throughout the 50 day incubation time. At both the 10 and 20 mM glucose concentrations, the disassembly rate is much slower, but with an apparent trend of glucose responsiveness for the two different polymer systems. The slow and/or low glucose responsiveness (*i.e.*, where higher glucose concentration is needed for significant response) is similar to previous reports on multilayered systems (26,53) and others (54–58) and is mostly attributed to an insufficient amount of phenylboronic acid moieties present in the system and/or to its weaker binding to glucose than, in this case, to PVA under physiological pH. The current results, however, are relevant for applications where longer sustained release is desired.

From the trends observed in Figs. 9, 10, and 11 for both PVA- and ChS-based films, the ABOL-BA-PAA-systems seem to more readily disassemble than DAB-BA-PAA films, or in other words, the latter are more stable. The trend is weak and is usually masked by the more dominant effect of the treatment (*i.e.*, reducing agents, pH, and glucose), but may be attributed to the difference between the primary amine and primary alcohol side chains. The extra positive charges brought in by the primary amines of DAB may help stabilize the layers (both with PVA and ChS), causing less effect against the various treatments, compared to primary alcohol side chains.

### COS-7 Cell Viability on Multilayered Systems

**Fig. 12:**
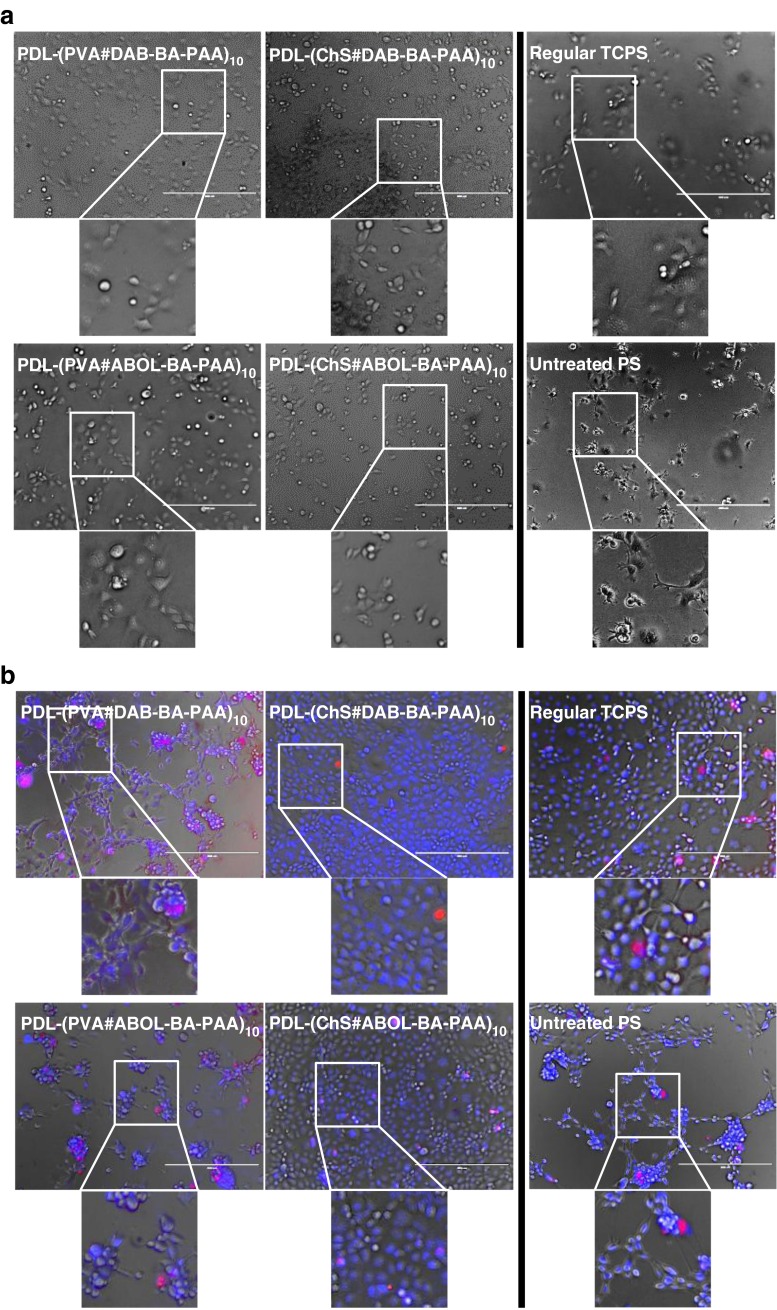
COS-7 (**a**) Light microscopy images of COS-7 cell after 6 h of culture on various surfaces. (**b**) Overlay fluorescence images following cell staining with Hoechst 33258 (*blue*, live) and EthD-1 (*red*, dead) after 2 days of culture on various surfaces. 10× magnification, bars = 400 μm.

In order to obtain a functionalized multilayered surface for biomedical applications, it is necessary to study the biocompatibility of the surface. For an *in vitro* experiment, the morphology and metabolic activity of cells cultured on the surface may serve as a preliminary indication. For this experiment, films were built directly on the surfaces of 96-well plates, and COS-7 cells were seeded directly on top of the films to emphasize the effect of the substrates on cell morphology and viability. At the end of the 2 days culture period, metabolic activity was measured. Figure 12a shows that at 6 h after cell seeding, all of the cells have attached. All of the COS-7 cells seeded on multilayers seem to have similar morphology to those seeded on regular commercial TCPS (tissue culture-treated polystyrene), *i.e.*, much better than the morphology of the cells seeded on the non-tissue culture treated polystyrene (*i.e.*, untreated PS, normally utilized for suspension culture). However, after 2 days of culture (Fig. 12b), COS-7 cells on PVA-based films are seen to aggregate, similar to those on untreated PS. The EthD-1 which stains for dead cells (red), however, shows no significant difference in the amount of dead cells. Therefore, to further confirm the results, metabolic activity assay was performed at the end of the 2 days culture experiment.

**Fig. 13:**
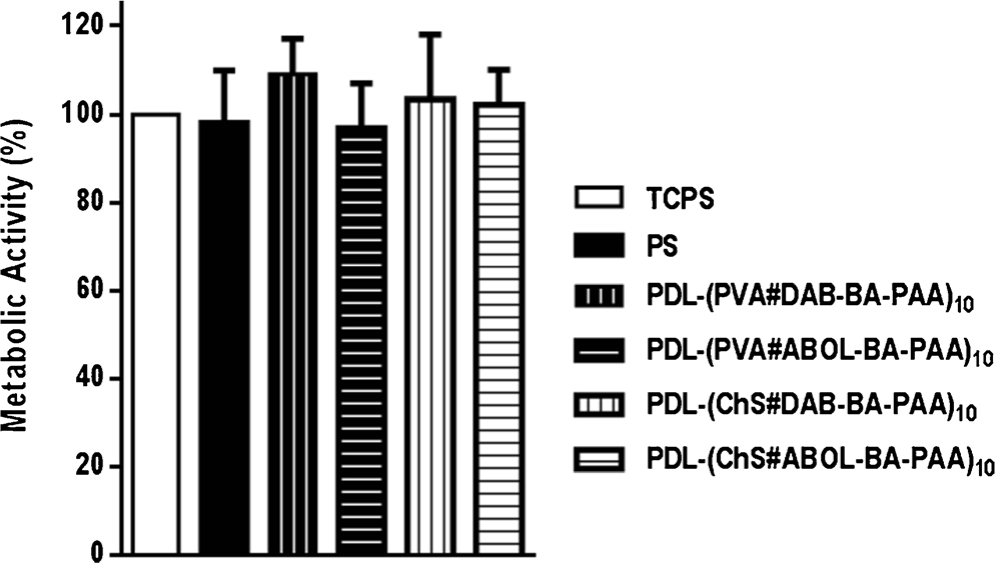
Metabolic activity of COS-7 cells cultured on untreated PS and various multilayered surfaces relative to TCPS control. All values are not significantly different from one another (*p* > 0.05).

In agreement with the live/dead staining results shown in Fig. 12b, the alamarblue (AB) assay for metabolic activity (Fig. 13) indicates that there is no significant difference in the metabolic activity of cells cultured on all of the studied surfaces. As expected, the more charged TCPS provides the best cell proliferation through faster cell attachment, while untreated PS provides slower cell attachment leading to slightly less metabolic activity. On the multilayered systems, ChS-based surfaces facilitate the best cell attachment, morphology and metabolic activity. Such high biocompatibility is expected given the natural biological origin of ChS. PVA-systems were observed to initially facilitate cell attachment, which means that the surface performs well in adsorbing anchoring proteins to facilitate cell attachment (59,60). However, upon longer culture duration the cells start to tend to aggregate, probably indicating slight cytotoxic effect of the surface. Considering the generally accepted biocompatibility of PVA (61), the effect may be induced by interaction of the cells with the BA-PAAs. Nevertheless the effect is mild, as the cells still show metabolic activity comparable to all the other surfaces. It can therefore be concluded that all of the multilayered systems investigated in this study are biocompatible.

## Conclusions

Two branched random copolymers of poly(amido amine)s containing phenylboronic acid functionality (BA-PAAs) have been synthesized and characterized for their possibility in forming multilayered thin films with PVA and ChS. The two BA-PAAs were ~50% functionalized with the same BA moiety, while the other 50% consisted of either primary amine (DAB) or primary alcohol (ABOL) side chains. The degree of branching was relatively low at ~10–30%, increasing overall molecular weight and possibly multilayer stability, while still facilitating aqueous-solublility at the multilayer assembly pH of 7.4.

Multilayer formation of the two BA-PAAs with both PVA and ChS were successful, with notable differences between PVA- and ChS-based systems. PVA films were thin, with ~100 nm thickness at 10 bilayer, while ChS films were at least 6 times thicker with possibly higher ChS content. The molecular weight of PVA was found to have no effect on the incremental deposition amount of materials. Furthermore, DAB-Bn-PAA, a control polymer without BA functionality, did not form multilayers with PVA, but with ChS multilayer formation did occur. This indicates that multilayer build-up of BA-PAAs with PVA is due to boronate ester formation. It was further found that PVA-based BA-PAA films are responsive to glucose, although with slow kinetics, indicating potential in drug delivery applications where sustained released is desired. In contrast, the positive multilayer formation by the control non-BA polymer (DAB-Bn-PAA) with ChS indicates the dominance of electrostatic interactions in facilitating multilayer formation between BA-PAAs and ChS. Consequently, these films were only found to be slightly responsive to glucose at the elevated concentration of 100 mM.

All of the films were responsive to various concentrations of glutathione and DTT albeit at different rates, owing to the presence of disulfide bonds in the BA-PAA polymer main chain. PVA-based films were found to be highly responsive to changes in pH with faster dissolution of the multilayers at lower pH. ChS-based films were also responsive to acidic pH but displaying much slower kinetics, and with a more complicated trend, *i.e.*, the disassembly rate was higher at pH 6 than at pH 4, albeit displaying the highest stability at pH 7.4. This trend is likely due to the change of ionization state of the protonable amines of BA-PAAs and the carboxylic acid groups of the ChS in the pH range of 4–6.

All of the films were found to be relatively biocompatible, with no significant cytotoxicity effect observed upon culturing COS-7 cells on top of the multilayers. ChS-based films notably provided cell morphology identical to tissue culture-treated PS. On PVA-based films, cells were found to attach well, but were found to slightly aggregate upon longer culture period, although no significant loss of viability was observed through both live/dead staining and metabolic activity assay.

The present study indicates the possibility of using the multilayers for biomedical applications, such as to provide drug releasing surfaces on stents and other implants. The different multilayers display different build-up profiles and properties due to the different underlying intermolecular interactions between the paired materials. For PVA-based films, the presence of the diol-repeating units in PVA can be utilized to incorporate small boronic acid-containing drugs into the films and provide release that is triggered by reducing agents, glucose, or acidic pH. For ChS-based films, in which part or the majority of the BA moieties of BA-PAAs are available for ester formation with guest molecules, the films can be exploited to incorporate diol-containing small molecules such as dopamine and provide its release through the triggers of reducing agents, and acidic pH. These two approaches are currently under investigation.

## Abbreviations

4AMPBA: 4-((4-aminobutylamino)methyl)phenylboronic acid
AB: AlamarBlue
ABOL: 4-aminobutanol
ABOL-BA-PAA: p(CBA-ABOL50%/4AMPBA50%)
BA: Boronic acid
BA-PAA: Phenylboronic acid-functional poly(amido amine)s
BnDAB: *N*-benzyl-1,4-butanediamine
CBS: Citrate buffered saline
ChS: Chondroitin sulfate
DAB: 1,4-diaminobutane
DAB-BA-PAA: p(CBA-DAB50%/4AMPBA50%)
DAB-Bn-PAA: p(CBA-DAB50%/BnDAB50%)
DCM: Dichloromethane
DMEM: Dulbecco’s Modified Eagle’s medium
LbL: Layer-by-layer
LFH: Laminar flow hood
NBDAB: *N*-Boc-1,4-diaminobutane
PDL-TCPS: Poly-D-lysine coated tissue culture polystyrene
PMMA: Poly(methyl methacrylate)
PS: Polystyrene
PVA: Poly(vinyl alcohol)
*t*BA: *tert*-butylamine
TEA: Triethylamine
TFA: Trifluoroacetic acid
